# Adherence to Antiplatelet Therapy after Coronary Intervention among Patients with Myocardial Infarction Attending Vietnam National Heart Institute

**DOI:** 10.1155/2019/6585040

**Published:** 2019-04-24

**Authors:** Ngoc Minh Luu, Anh Tuan Dinh, Thi Thu Ha Nguyen, Van Huy Nguyen

**Affiliations:** ^1^Department of Biostatistics and Medical Informatics, Institute for Preventive Medicine and Public Health, Hanoi Medical University, 01 Ton That Tung Str, Dong Da Dist, Hanoi, Vietnam; ^2^Vietnam National Heart Institute, 78 Giai Phong Str, Phuong Dinh Precinct, Dong Da Dist, Hanoi, Vietnam; ^3^Department of Health Management and Organization, Institute for Preventive Medicine and Public Health, Hanoi Medical University, 01 Ton That Tung Str, Dong Da Dist, Hanoi, Vietnam

## Abstract

Adherence to antiplatelet therapy is critical to successful treatment of cardiovascular conditions. However, little has been known about this issue in the context of constrained resources such as in Vietnam. The objective of this study was to examine the adherence to antiplatelet therapy among patients receiving acute myocardial infarction interventions and its associated factors. In a cross-sectional survey design, 175 adult patients revisiting Vietnam National Heart Institute diagnosed with acute myocardial infarction were approached for data collection from October 2014 to June 2015. Adherence to antiplatelet therapy was assessed by asking patients whether they took taking antiplatelet regularly as per medication (do not miss any dose at the specified time) for any type of antiplatelet (aspirin, clopidogrel, ticlopidine...) during the last month before the participants came back to take re-examinations. The results indicated that the adherence to antiplatelet therapy among patients was quite high at 1 month; it begins to decline by 6 months, 12 months, and more than 12 months (less than 1 month was 90.29%; from 1 to 6 months 88.0%, from 6 to 12 months 75.43%, and after 12 months only 46.29% of patients). Multivariable logistic regression was utilized to detect factors associated with the adherence to antiplatelet therapy. It showed that patients with average income per month of $300 or more (OR=2.92, 95% CI=1.24-6.89), distance to the hospital of less than 50km (OR=2.48, 95% CI: 1.12-5.52), taking medicine under doctor's instructions (OR=3.65; 95% CI=1.13-11.70), and timely re-examination (OR=3.99, 95% CI=1.08-14.73) were more likely to follow the therapy. In general, the study suggested that to increase the likelihood of adherence to antiplatelet therapy it is important to establish a continuous care system after discharging from hospital.

## 1. Introduction

Coronary intervention in acute myocardial infarction treatment has been widely applied around the world, although its long-term effects can be limited by the occurrence of reinfarction, stent thrombosis, and restenosis in intravascular interventions. However, these problems can be prevented using anticoagulants, antiplatelet drugs with adequate doses [1]. Dual antiplatelet therapy (aspirin and clopidogrel) after coronary intervention has been a standard approach to prevent secondary cardiovascular risk. Failure to abide by the treatment was closely related to a doubled risk of myocardial infarction, four times increase in risk of acute coronary syndrome, and twice the cost for hospitalization and treatment [2]. Despite its critical role, previous studies showed that this phenomenon was controlled only during the time the patient stayed in the hospital; after hospital discharge, the rate of continuing to take the drugs after the medical intervention was relatively low [3, 4].

A number of factors associated with nonadherence to antiplatelet therapy have been examined by prior studies. They include lower education level, immigration status, poor awareness of antiplatelet drugs, high drug costs, lack of understanding about medical conditions or the value of adherence to treatment, ethnic minorities, elderly, side effects of drugs, ineffective treatment, financial reasons, medication beliefs, and effects of herbal medicine [3, 5–8]. Although numerous prior studies have been conducted in other countries, most focused on patients after coronary stenting [9]. In Vietnam, there has been little research on adherence to antiplatelet therapy among patients with myocardial infarction. The objective of this study was to examine the adherence to antiplatelet therapy among patients with acute myocardial infarctions and associated factors who attended a central level cardiology center.

## 2. Methods

### 2.1. Research Design and Location

A cross-sectional study has been used to identify the factors related to conformity with taking antiplatelet theray in patients. Data were collected using structured questionnaires in the Vietnam Heart Institute, Bach Mai Hospital, in Hanoi, Vietnam.

### 2.2. Sample Size and Participants

This study came part of a small project conducted during October 2014 and June 2015. The total sample of the study was 175 persons, including all acute myocardial infarction patients over 18 years of age who attended the clinic and received coronary artery intervention in the Vietnam National Heart Institute from October 1st, 2014, to June 30th, 2015. Most patients had stable angina; percutaneous coronary intervention (PCI) was applied for all patients and not all cases were stented. PCI is an indication of acute MI. Patients were excluded from the study if they declined to participate, had an associated health condition such as neurological disease, dementia, or other mental health problems, and if they did not revisit for health check as appointed by a physician during our data collection period.

### 2.3. Measures

A questionnaire was designed based mainly on research variables. The questionnaire was drafted and tested on a sample of 30 patients who attended and received treatment after coronary intervention at the Vietnam National Heart Institute in September 2014. After the pilot testing, the questionnaire was adjusted. It comprised the following variables.

The* dependent variable* was adherence to antiplatelet therapy during the last month before the participants came back for the re-examinations. Adherence during the past month was defined as the status of patients taking the antiplatelet regularly and constantly (not forgetting to take any dose at the specified time) for any type of antiplatelet medication (aspirin, clopidogrel, ticlopidine,...) within last month after patients had coronary artery intervention and after leaving the hospital. No adherence over the last month was defined as the patients not taking or forgetting to take the drug at least once for any type of antiplatelet (aspirin, clopidogrel, ticlopidine,...) within one month before they came back for the re-examinations (since interview date).

*Independent Variables*.* Demographic variables* included age (31-60/≥ 60), sex (female/male), occupation (farmer, informal worker/officer, retired, and student), education level (intermediate, high school or lower/college, university, or over), area, and income (≥ 6.000.000VND/<6.000.000VND) (≥ $272/<$272)

*Access and behavior variables* comprised a number of variables: distance to hospital (<50km/ ≥ 50km), medicine intake following doctors' instructions (irregularly/regularly), knowledge of drug effect (do not know/know), knowledge of time to take medicine (do not know/know), knowledge of consequence when forget to take medicine (do not know/ know), re-examination (on time/not on time), knowledge what to do when forget to take medicine (don't know/ know), understanding the doctors' explanation (understand ≥50%/understand <50%), what to do when forget to take medicine (double dose on next day/continue take medicine at usual doses and report to doctor/contact doctor/do not continue to take medicine), knowledge of cause of disease (hereditary/lifestyle/other), identifying acute myocardial infarction before going to hospital (yes/no), and having consulted doctor before surgery and before leaving hospital (yes/no); proportion of patients adhering to the antiplatelet over the treatment time (over 6 months, 6-11 months, 12-23 months, and > 24 months).

### 2.4. Data Analysis

Data collection was conducted by residents at the Institute, trained to do the interviews. Patients were interviewed when they revisited the Institute for a health check following an appointment with a physician. After their examination, they were approached for data collection. Before each interview, they were informed about research content and objectives and about how the interview data would be documented and reported and that their confidentiality would be respected. The study did not record the names of participants. Data gathered were treated as confidential and accessible only to the principal investigator and his research team. Each interview took 25 to 30 minutes. The data collection ended within nine months, from October 1st, 2014, to June 30th, 2015.

Data were entered into the computer with EPI DATA 3.1 and then transferred to Stata 11.1 for analysis. Descriptive statistics (number, proportion, mean, and standard deviation) were used to describe the adherence to antiplatelet therapy among patients with acute myocardial infarction who attended and had an intervention at Vietnam National Heart Institute. Chi-squared test and/or Fisher's exact test was employed to compare differences between proportions. A multivariable logistic regression model was utilized to detect the factors associated with the adherence to antiplatelet treatment. The associations were assessed with odds ratios and 95% confidence intervals. The model fit was assessed with the criteria of Hosmer and Lemeshow (P-value of model fit *χ*2 >0.05, P-value of model coefficients<0.05, and Nagelkerke's* R*^2^ is quite large).

### 2.5. Research Ethics

This study was scientifically and ethically reviewed and approved by the Scientific Panel of the Institute for Preventive Medicine and Public Health, Hanoi Medical University, at Decision Number 268/QĐ-ĐHYHN on Febuary 12^th^, 2015; it was also approved by the Vietnam National Heart Institute.

## 3. Results

### 3.1. Characteristics of the Participants

A total of 175 participants were surveyed. The majority of patients were old (≥ 60 years of age, 65.14%). Most of the subjects were male (72.99%) and were ethnically Kinh (98.85%). As to educational level, while 80.57% of the patients had high school level, the percentage of patients with undergraduate and postgraduate education level was relatively high (15.43%). Regarding occupation, more than 50% were retired, followed by farmers (21.14%); 63.43% of the subjects had an average income per month lower than 6 million VND (about 272 USD).

### 3.2. Adherence to Antiplatelet Therapy

Adherence to antiplatelet therapy is described in Table 1. Most patients never missed taking their medicine (70.86%). The majority of the patients who forgot their medicine called the doctor immediately (94.12%). The proportion of those who returned for the doctor's appointment was 88.57%, while 95.43% had sought disease-related information before coming to the hospital. Almost all of them (95.43%) sought consultation with a doctor before surgery and again before leaving the hospital and 87.43% of the sample understood more than 50% of the doctors' explanations. However, 50.3% had incorrect beliefs about myocardial infarction.

**Figure 1:**
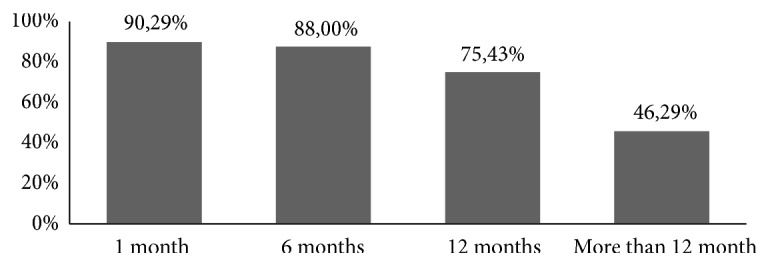
Trend of adherence to antiplatelet therapy over the time.

Figure 1 showed the trend of adherence to antiplatelet therapy. As can be seen, the trend of adherence declined over time after discharge from the hospital, from 90.29% within less than 1 month, 88% from one to six months, 75.43% six to 12 months, and only 46.29% after 12 months.

### 3.3. Factors Associated with Adherence to Antiplatelet Therapy

Table 2 shows the results of univariate analysis on the relationship between factors with the adherence to antiplatelet therapy. Only two factors were significantly associated with adherence to medication: closer distance to hospital (<50km) and knowing the effect of medication. The results of multivariable logistic regression are presented in Table 3. Four variables were significantly associated with adherence to therapy: average income per month (≥ 6.000.000VND: OR=2.92, 95%CI=1.24-6.89), closer distance to the hospital (<50km: OR=2.48, 95%CI=1.12-5.52), taking medicine following the instructions (regularly follow the instructions: OR=3.65; 95%CI=1.13-11.70), and timely re-examination (on time: OR=3.99, 95%CI=1.08-14.73). The multivariable model (*χ*2=28.4, p<0.05) explained 14% of variance (Nagelkerke's R2=0.14). This model satisfied Hosmer-Lemeshow goodness-of-fit standards (*χ*2= 53.78; p = 0.33).

## 4. Discussion

The cessation of adherence to antiplatelet therapy is known to be an important predictor of poor outcomes after coronary stenting. This study showed that the proportion of patients from the Vietnam National Heart Institute complying with antiplatelet therapy was 70.86% during the past month. Regarding the trend of adherence to antiplatelet treatment over time, we found a gradual decrease with longer treatment times, declining from over 90% in the first month to less than 50% after one year. This outcome was consistent with previous studies, despite the differences in other factors including income and health care systems between our sample and those from US [10–12], Ko et al. in Canada, and Ikari in Japan in 2009 [13]. However, other reports demonstrated a stable or only slightly decreased rate of compliance at longer treatment times [14–17]. These studies, where the rate of compliance at three times points (1, 6, and 12 months) fluctuated around 90%, were usually from developed countries, mostly the United States. One of the possible reasons for their success was the effectiveness of a clinical guidelines update for percutaneous coronary intervention in 2017 [18] to increase adherence [3]. In addition, in these countries, patients had better economic conditions, higher income, better education, more coverage of health insurance, and good relationships with physicians. There was good follow-up after surgery as well as better understanding of the disease compared with developing countries, both of which may contribute to keeping adherence rates stable over time. In Vietnam, currently, the application of the abovementioned guidelines to treatment may be not good, resulting in poor adherence to medication. Other factors that may reduce compliance rates over time are the low income, lack of knowledge about the importance of drug use, and lack of communication between doctors and patients. Therefore, better connections between patients and physicians, accompanied by good explanation of the doctors about the consequences of noncompliance, may be necessary to maintain a consistent compliance rate over the longer time period.

*Factors Associated with the Adherence of Antiplatelet*. The univariate analysis showed that adherence to antiplatelet treatment after coronary intervention was significantly related to the distance from home to the hospital and to the patient's knowledge. Firstly, being nearer to the hospital (<50 km) increased the proportion of adherent patients by 2.74 times. The multivariate model also provided statistically significant results on the association of distance to the hospital with adherence to the drug. It can be predicted that patients living far from the hospital had less chance to access medical services, had more difficulties in seeking medical care and had limited contact with doctors and health care workers, while the contact and connection between physicians and patients and follow-up after discharge from hospital have been shown to have a major influence on treatment adherence [19, 20]. On the other hand, our study also showed that when patients knew the effecst of the drug they were 2.74 times more likely to maintain adherence. For example, Blinch et al. also reported that a lack of explanation by health workers about the usefulness of taking medication when the patient is discharged from hospital corresponded to a higher rate of noncompliance [19]. Clearly, health education and health communication must be implemented in an effective way to support compliance.

In our multivariate analysis, we identified three additional independent variables associated with the patient compliance: income, taking medicine, and coming for re-examination. Firstly, adherence was 2.92 time higher in patients with income greater than 6.000.000VND (about 272 USD). This finding was consistent with Ko's study in Japan, where patients co-pay with insurance, so low-income people faced a challenge to prolong compliance [13]. Unlike in Japan, in Vietnam, according to the Ministry of Health, the insurance covers both aspirin and clopidogrel [21]. However, the coverage of health insurance in Vietnam was only about 70% in 2015, and out-of-pocket payment were still relatively high [22], leading to high payments for poor patients. To address this problem, increasing health insurance coverage is needed to reduce the burden for low-income patients, which would improve their adherence. According to the study results, the more patients took medication as directed by the doctor regularly and came for re-examination on time, the more they were adherent. This suggests that if patients are aware of their condition and the extent of the risks (of re-narrowing), the commitment to compliance is higher. Through this analysis, we clarified that the information shared between the doctor and the patient plays a very important role in adherence to therapy as well as its effectiveness. The systematic review by Matthew found that lower education level, immigrant status, and a lack of clearly-communicated instructions regarding DAPT prior to discharge from the index hospitalization were associated with non-adherence [3], which quite similar our findings.

Through the above logistic regression analysis, two statistically significant dependent variables in univariate analysis were the distance and the knowledge of the effect of the drug. Meanwhile, in the multivariate model, three additional factors that were statistically significant were re-examination status, monthly income, and taking medicine as directed by the doctor regularly; however the knowledge of the patient was not statistical significance anymore. This can be predicted that the association between this variable and the adherence may be affected by confounding factors so that, in multivariate analysis, this variable is no longer statistically significant with the compliance of antiplatelet. Furthermore, we can predict that this is due to the influence of an intermediate variable that is difficult or impossible to control in univariate analysis. So, when introduced into multivariate analysis, three completely different variables appeared with statistical significance. In this case, re-examination status, monthly income, and taking medicine as directed by the doctor regularly were both effect of adherence but this affect only occurred when the variables were present at the same time (resonance).

Through the above results, adherence to treatment may depend to a great extent on the contact between the doctor and the patient. An adequate explanation in hospital before discharge and repetition about adherence when the patients come for re-examination will help to improve the patient's knowledge and behavior on adherence to treatment. The distance and income factors can be barriers that limit not only knowledge but also coming for re-examination and seeing a doctor. Providing information in other ways, such as giving specific instructions and reminders by phone or messages for patients living far from the hospital might be an effective solution. Encouraging people to return to hospital to take examination regularly and to use health insurance to reduce the burden of medical expenses is also suggestions to increase the level of compliance.

## 5. Limitations of the Study

This study had several limitations. Firstly, because of the low number of patients who revisited the hospital for seven consecutive months, the sample size was not as high as expected. Most of the participants were patients with the appointments for re-examination, which may not be representative for the entire target population of patients at the National Heart Institute. As some of the questions asked about the respondents' previous practices, recall bias might occur. To reduce this kind of bias, we have chosen a time interval of only the past month when asking the respondents about the compliance of antiplatelet therapy. The cross-sectional design means that temporal relationship between the factors and the adherence to antiplatelet therapy cannot be determined. In spite of these limitations, we could find significant associations with factors related to adherence which were comparable to those reported in other similar situations.

## 6. Conclusions

The results of this study could provide important evidence to inform better clinical practice and health care in Vietnam. The percentage of antiplatelet adherence of acute myocardial infarction patients in this study tended to decrease over the treatment period. Distance from home to the hospital, knowledge of the patient about the correct treatment, and income were the main factors that affected adherence. Enhancing interaction between physicians and patients is essential to improving patients' knowledge as well as for effective treatment. Contact with the patient and telephone or text reminders about taking medicine should be considered as interventions to reduce nonadherence. Other studies using other methods, such as longitudinal studies, intervention research, and qualitative research, are needed to identify other factors related to nonadherence for a more comprehensive view.

## Figures and Tables

**Table 1:** Adherence to antiplatelet therapy.

Variables (n = 175)	n (%)

*Adherence to medication during a last month *	
Adherence	124 (70.86)
No adherence	51 (29.14)
*Actions when forgot medicine (n=51)*	
Did not forget	124 (70.86)
Double dose on next day	0 (0.00)
Continue to take medicine at usual doses and report to doctor	1 (0.57)
Contact doctor	47 (26.86)
Do not continue to take medicine	2 (1.14)
*Re-examination *	
By appointment	155 (88.57)
Not by appointment	20 (11.43)
*Seeking information on myocardial infarction before visiting hospital *	
Yes	167 (95.43)
No	8 (4.57)
*Knowledge of disease cause *	
Genetics	84 (50.30)
Lifestyle	48 (28.74)
Others	35 (20.96)
*Having consultation with a doctor before surgery and before leaving hospital *	
Yes	167 (95.43)
No	8 (4.57)
*Understanding the doctors' explanation *	
<50%	22 (12.57)
≥50%	153 (87.43)

**Table 2:** Univariate logistic regression of factors associated with adherence to therapy.

Variables	OR (95%CI)

*Age group*	
≥ 60	1
31 – 60	1.40 (0.63-3.09)
*Sex*	
Female	1
Male	1.26 (0.56-2.82)
*Education level*	
Intermediate, high school or lower	1
College, university or higher	1.60 (0.76-3.36)
*Occupation*	
Farmer, informal worker	1
Officer, retired, student	1.9 (0.84-4.27)
*Income per month ($1≈22,000VND)*	
≥ 6.000.000VND (≥*$272)*	1
> 6.000.000VND (<*$272)*	1.13 (0.53-2.41)
*Distance to hospital*	
≥ 50km	**1**
< 50km	**2.74 (1.15-6.64)**
*Taking medicine*	
Irregularly follow the instructions	1
Regularly follow the instructions	1.07 (0.37-3.10)
*Knowledge of medicine effect*	
Don't know	**1**
Know	**2.74 (1.23-6.11)**
*Knowledge of time to take medicine*	
Don't know	1
Know	2.02 (0.88-4.67)
*Knowledge of consequences when forgetting to take medicine*	
Don't know	1
Know	2.90 (0..95-8.90)
*Re-examination*	
Not on time	1
On time	1.53 (0.42-5.58)
*Knowledge to handle when forget to take medicine*	
Don't know	1
Know	2.37 (0.91-6.17)
*Understanding the doctors' explanations*	
≤ 50 %	1
>50 %	1.54 (0.55-4.28)

**Table 3:** Multivariate logistic regression of factors associated with adherence to therapy.

Variables	Adherence	AOR∗ (95% CI)
	%	

*Income per month*		
< 6.000.000	78.13	1
≥ 6.000.000	80.18	2.92 (1.24 – 6.89)
*Distance to hospital*		
≥ 50km	73.58	1
< 50km	88.41	2.49 (1.12 – 5.52)
*Taking medicine*		
Irregularly follow the instructions	79.33	1
Regularly follow the instructions	78.26	3.65 (1.13 – 11.70)
*Re-examination*		
Not on time	78.71	1
On time	85.00	3.99 (1.08-14.73)
*Model indicators*		
*χ*2 and p-value of model coefficients	*χ*2 =28.40; p ≤ 0.001	
*χ*2 and p-value of Hosmer and Lemeshow	*χ*2= 53.78; p = 0.3316	
Adjusted R^2^	0.14	

∗AOR=Adjusted Odds Ratio.

## Data Availability

The data used to support the findings of this study are available from the corresponding author upon request.
